# Pingyin rose essential oil alleviates LPS-Induced inflammation in RAW 264.7 cells via the NF-κB pathway: an integrated in vitro and network pharmacology analysis

**DOI:** 10.1186/s12906-022-03748-1

**Published:** 2022-10-14

**Authors:** Rifat Nowshin Raka, Ding Zhiqian, Yuan Yue, Qiao Luchang, Park Suyeon, Xiao Junsong, Wu Hua

**Affiliations:** 1 Beijing Engineering and Technology Research Center of Food Additives, College of Food and Health, Beijing Technology and Business University, Building No. 8, Fucheng Road 11#, Haidian District, (BTBU), Beijing, 100048 China; 2 College of Chemical and Materials Engineering, Beijing Technology and Business University, Building No. 1, Fucheng Road 11#, Haidian District, Beijing, 100048 China

**Keywords:** *Rosa rugosa* cv. *Plena*, Essential oil, Anti-inflammation, Oxidative stress, NF-κB signaling, Network Pharmacology

## Abstract

**Background:**

*Rosa rugosa* cv. Plena, a cultivar of *Rosa rugosa,* has a history of more than 1300 years of application in both medicine and food in China. The essential oil of *Rosa rugosa* cv. Plena (PREO) is one of the most frequently used additives in food, cosmetics and aromatherapy. PREO exhibits some anti-inflammation, antioxidant and nerve alleviating effects. However, the mechanisms behind these effects are still unclear.

**Methods:**

The composition of PREO was determined by GC‒MS. Network pharmacology was performed to predict the possible compound-target network and analyze the possible targets against inflammation and oxidative stress. An inflammatory immune cell model was constructed by exposing RAW 264.7 cells to LPS. A series of experiments, including biochemical assays, RT‒PCR, and western blotting, were conducted to investigate the anti-inflammatory and antioxidative effects of PREO.

**Results:**

PREO treatment significantly (*p* < 0.05) alleviated inflammatory and oxidative biomarkers such as NO, ROS, and MDA and preserved SOD and CAT activities. GC‒MS analysis revealed that PREO consists of 57 compounds, mainly monoterpenoids. Network pharmacology revealed that citronellol, farnesol, ethyl octanoate, geranyl acetate, and methyl eugenol were active components interacting with several inflammatory pathway proteins. By measuring the gene and protein expression of possible targets by qRT‒PCR and western blotting, PREO anti-inflammatory responses in LPS-treated RAW 264.7 cells might be associated with the regulation of NF-κB signaling. Molecular docking showed that PREO components can interact with different proteins involved in the NF-κB pathway.

**Conclusion:**

The integrated study of molecular analysis and network pharmacology suggested that PREO might be a potential anti-inflammatory agent to treat inflammation and oxidative stress.

## Highlights

➢ PREO is a mixture of aromatic components, mainly monoterpenoids.

➢ PREO reduces the expression of proinflammatory cytokines and oxidative stress markers.

➢ Network pharmacological studies indicated that PREO might downregulate inflammation through the NF-κB  signaling pathway, and these results were confirmed by Western blot investigation.

➢ Molecular docking analysis revealed the potential of these compounds as possible ligands.

**Graphical Abstract:**

## Graphical abstract

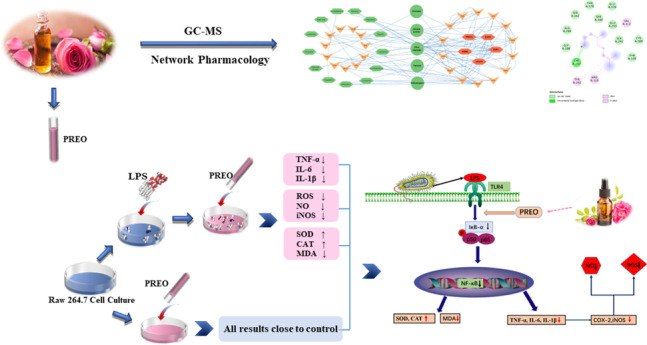

## Background

Inflammation is a defensive response of the body against stimulation by chemical substances, physical factors, and microbial pathogens [1]. Short-term inflammation helps to improve and promote the body’s resistance. However, uncontrolled long-term inflammation can ravage organs, develop into chronic inflammation, and trigger diseases, such as rheumatoid arthritis, type-2 diabetes, and inflammatory bowel disease [2]. Macrophages are immune cells that play significant roles in host defenses and tissue homeostasis through phagocytosis, cytokine secretion and antigen presentation to T cells [3]. When macrophages are activated by gram-negative bacterial endotoxin lipopolysaccharides (LPS), Toll-like receptors (TLRs), especially TLR4, sense them and activate the production of inflammatory mediators (NO, PEG2) and cytokines (IL-1β, IL-6 and TNF-α) through several pathways, such as nuclear factor-kappa B (NF-κB) and mitogen-activated protein kinase (MAPK) signaling. In addition, excessive ROS play a crucial role in increasing oxidative stress, amplifying inflammation, and causing deleterious consequences [4–6]. Scientists have found that NF-κB dimers are responsible for the production of inflammation-related growth factors and cytokines. As a heterodimer composed of p50 and p65, NF-κB dimers will not be activated if any subunit loses function [7]. The phosphorylation-induced proteasomal degradation of inhibitors of NF-κB proteins, such as IκB-α, is very important for inducible NF-κB activation and inflammatory regulation. Most drugs are designed to target NF-κB signal transduction to find an anti-inflammatory solution. Therefore, how to prevent and reduce excessive inflammation as well as oxidative stress has become major focus of attention. However, nonsteroidal anti-inflammatory drugs (NSAIDs), which are popular options to treat inflammation, often cause adverse side effects [8]. A great number of studies have indicated that plant extracts, such as phytochemicals and essential oils, have strong anti-inflammatory or antioxidative effects with very mild to no side effects [9–13].

Rose essential oil (REO) is extracted from fresh rose flowers. It has a variety of biological effects, such as anti-inflammatory and analgesic effects, whitening and moisturizing, promoting the metabolism and regeneration of epidermal cells, and inhibiting free radical activation [14–16]. Different rose species have different genetic backgrounds and metabolism systems, the composition of REO can vary, and their bioactivities would therefore differ greatly [17, 18]. Most of the scientific investigations around REO focused on the composition and bioactivities of *Rosa damascena*, the most widely applied and effective rose species in the world [19]. In China, *Rosa rugosa* Thunb. was recorded as a prescribed medicine for blood circulation and liver nourishment in the Chinese Pharmacopoeia 2015 [20–22]. For over 1300 years, *R. rugosa cv.* Plena, a cultivar of *R. rugosa* Thunb., has been cultivated uniquely in Pingyin town in Shandong Province in China, and its buds have been used as a very exclusive traditional Chinese medicine (TCM) [22]. Pingyin rose essential oil (PREO) obtained from fresh flowers of *R. rugosa cv.* Plena. has been used by local people as a food and cosmetic additive due to its rich fragrance. It has also been effectively used to treat inflammation and antioxidants for a long time. However, there have been few reports on the bioactivity of PREO, and the underlying mechanisms of its anti-inflammatory and antioxidative effects have not yet been explored.

Therefore, in this study, we tried to identify the possible inflammatory pathways in which PREO may participate using network pharmacology approaches. Then, the anti-inflammatory effect was evaluated, and the possible pathway proposed by network pharmacology was verified using an LPS-induced inflammation model in RAW 264.7 cells. Molecular docking was performed to identify the potential compounds with anti-inflammatory effects in PREO.

## Materials and methods

### Chemical reagents

PREO was donated by Jinan Wanfeng Rose Products Co., Ltd. from Pingyin, Shandong Province, China. The plant source was *Rosa rugosa* cv. *Plena* cultured in Shandong Province. Dulbecco's modified *Eagle’s* medium (DMEM) and fetal bovine serum (FBS) were purchased from GIBCO BRL (Grand Island, NY, USA). Sodium pyruvate was purchased from Solarbio Life Sciences (Beijing, China). LPS (*Escherichia coli* O127:B8) and 20,70-dichlorofluorescein-diacetate (DCFH_2_-DA) were purchased from Sigma‒Aldrich (St. Louis, MO, United States). The MTT assay kit, bicinchoninic (BCA) protein assay kit, total nitric oxide (NO) assay kit, catalase (CAT) assay kit, superoxide dismutase (SOD) assay kit, and malondialdehyde (MDA) assay kit were obtained from Beyotime Institute of Biotechnology, Ltd. (Shanghai, China). Rabbit monoclonal antibodies against IκB-α, p-IκB-α, p65, p-p65, p50, and p-p50 and a mouse monoclonal antibody against β-actin were purchased from Cell Signaling Technology (Danvers, MA, United States). Rabbit monoclonal antibodies against iNOS and COX-2 were purchased from Abcam (Cambridge, UK). The polymerase chain reaction (PCR) primers for β-actin, TNF-α, IL-1β, IL-6, and iNOS were acquired from Beijing Genomics Institute (BGI) (China). An RNA extraction kit was purchased from Transgen Biotech Co Ltd. (Beijing, China). The RT‒PCR kits were obtained from Toyobo, Japan. High-sig ECL Western Blotting Substrate was obtained from Tanon™ (Tanon, Shanghai, China). Other chemicals used in this study were of analytical grade and purchased from Beijing Chemical Works (Beijing, China).

### Cell culture

RAW 264.7 macrophage cells were obtained from the Stem Cell Bank, Institute of Zoology (China Academy of Sciences, Beijing). Dulbecco's modified Eagle’s medium (DMEM) supplemented with 10% FBS, 1% glutamine, and 1% sodium pyruvate was used as the basic cell culture medium. Unless otherwise noted, all the cells were cultured at 37 °C in an incubator simplified with 5% CO_2_.

### Cell viability assay

Approximately 100 μL of RAW 264.7 cell suspension (2 × 10^5^ cells/mL) was seeded in each well of the 96-well plate (0.32 cm^2^) and incubated until confluency. Then, the culture medium was replaced with serum-free medium containing LPS and different doses of PREO, and the plate was incubated under the same conditions for another 20 h. Then, the culture medium was removed, and 10 µL of MTT (5 mg/mL) was added to each well, followed by incubation for 4 h. Subsequently, 10% SDS was added to each well, and the plate was incubated for another 12 h. Finally, the absorbance at 570 nm was measured on a microplate reader (Tecan, Männedorf, Switzerland).

$$\mathrm{Cell\ viability }\left(\mathrm{\%}\right)=\frac{\mathrm{Absorbance\ of\ sample}}{\mathrm{Absorbance\ of\ control}} \times 100\%$$

### Determination of nitric oxide level and intracellular ROS scavenging activity in RAW 264.7 macrophage cells

Approximately 100 μL of RAW 264.7 cell suspension (2 × 10^5^ cells/mL) was seeded in the wells of a 96-well plate and incubated. After the cells reached confluency, the medium was replaced with serum-free medium containing LPS and different doses of PREO. Twenty hours after incubation in the same environment, 60 µL of supernatant was mixed with Griess reagent according to the kit instructions. After incubation at room temperature for 10 min, the absorbance at 540 nm was measured on a microplate reader (Tecan, Männedorf, Switzerland).

For ROS measurement, the same volume of cell suspension was seeded in the wells of a 96-well plate, cultured and treated with LPS as mentioned above. Then, the medium was replaced by DCFH-DA (10 μmol/mL) and incubated for 30 min at 37 °C and 5% CO_2_. After that, the medium was discarded, and the wells were washed twice with PBS. Then, 200 μL of serum-free medium was added to each well, and the fluorescence intensity was measured with an excitation wavelength of 485 nm and an emission wavelength of 535 nm using a spectrophotometer (Tecan, Männedorf. Switzerland).

### Determination of MDA, SOD, and CAT levels in LPS-treated RAW 264.7 macrophage cells

RAW 264.7 cells (3 mL) were seeded in Petri dishes (d = 3.5 cm) at a density of 1.6 × 107/mL and incubated until reaching confluency. The culture medium was then replaced with serum-free medium containing LPS and various doses of PREO, and the plate was cultured for another 20 h under the same conditions. RAW 264.7 cells were washed twice with cold PBS and harvested by a cell scraper. Then, cell lysis fluid was added to lyse the cells, and the supernatants of lysed cell centrifugation (10,000 g at 4 ℃ for 5 min) were collected. The MDA, SOD, and CAT activities were determined by using commercial kits (Nanjing Jiancheng Institute of Bioengineering, Nanjing, China).

### Quantitative real-time polymerase chain reaction (qRT‒PCR)

RAW 264.7 cells (3 mL) were plated in a petri dish at a density of 1.6 × 10^7^/mL and incubated until they reached confluency. The culture medium was then exchanged with serum-free medium containing LPS and different PREO concentrations, and the plate was cultivated for another 20 h under the same conditions. Cells were harvested as described in Sect. 2.5. Then, total RNA from RAW 264.7 cells was extracted using TRIzol reagent following the manufacturer's instructions. The 260/280 ratio of RNA was determined using a q3000 spectrophotometer. The RNA was then reverse-transcribed using ReverTra Ace qPCR RT master mix with gDNA remover (Toyobo, Japan). Approximately 0.5 µg of total RNA from the cells was used for RT‒PCR with a Bio-Rad CFX96 touch system using SYBR Green master mix (Toyobo, Japan). The expression levels of TNF-α, interleukin-1β (IL-1β), IL-6, and iNOS were quantified using β-actin as an internal control. The primer sequences are presented in Table 1. Target gene quantification was analyzed by comparison with the β-actin gene and calculated following the 2^−△△CT^ method [23].

**Table 1:** Primer sequences used in RT‒PCR

Gene	Primer Sequences
*β-Actin*	F: CCTAGAAGCATTTGCGGTGCACGATG
	R: TCATGAAGTGTGACGTTGACATCCGT
*TNF-α*	F: TACAGGCTTGTCACTCGAATT
	R: ATGAGCACAGAAAGCATGATC
*IL-1β*	F: TGCAGAGTTCCCCAACTGGTACATC
	R: GTGCTGCCTAATGTCCCCTTGAATC
*IL-6*	F: AAGTGCATCATCATCGTTGTTCATACA
	R: GAGGATACCACTCCCAACAGACC

### Gas chromatography‒mass spectrometry analysis of PREO

PREO was analyzed on an Agilent 6890 gas chromatograph equipped with an Agilent 5973 mass selective detector (Agilent Technologies, Folsom, CA). The column was a DB-WAX Ultra Inert column (30 m × 0.25 mm, 0.25 µm). The column temperature was programmed as follows: 50 °C for 1 min, 250 °C at 10 °C/min, and finally 250 °C for 5 min. The injection port temperature was 260 °C, while the detector temperature was 250 °C. The carrier gas was helium with a flow rate of 1 mL/min. The injection volume was 1 μL. The MS conditions were as follows: ionization voltage, 70 eV; ion source temperature, 150 °C; and mass spectra acquired over the mass range of 50–550 m/z. Compounds in PREO were identified by comparison to the mass spectra and retention indices in the NIST mass spectral library.

## Network pharmacology

### Compound target network

All the components identified in GC‒MS analysis were searched under the “chemical name” in the TCMSP (Traditional Chinese Medicine for System Pharmacology database and analysis platform (TCMSP, https://tcmspw.com/tcmsp.php), and target prediction was performed for selected compounds with the Swiss Target prediction (http://www.swisstargetprediction.ch/) webtool. “*Homo sapiens*” genes were documented from the GeneCards platform using the keywords “Anti-Inflammation” (AI) and “Anti-oxidation” (AO). The Venny 2.0 online tool was used to determine genes shared in common by PREO targets, AI, and AO. Webgestalt and Kobas 3.0 were used to perform analysis based on Gene Ontology (GO) and KEGG enrichment. The protein‒protein interaction (PPI) network of common genes was obtained from the STRING database. Then, a compound-target network was constructed with Cytoscape, and the top nodes were identified depending on their degree and closeness.

### Western blot analysis

Three-milliliter aliquots of RAW 264.7 cells were cultured in petri dishes (d = 3.5 cm) at a density of 1.6 × 107/mL and treated as described in Sect. 2.6. Then, they were collected, and a cell lysis buffer containing protease inhibitors or phosphatase inhibitors was added following the kit instructions. Then, the solution was kept on ice for 30 min and later centrifuged at 3000 rpm for 15 min at 4 °C. The supernatants were separated, and protein concentrations were quantified by using a BCA protein assay following the microplate procedure. Thirty micrograms of protein from each sample was separated by sodium dodecyl sulfate‒polyacrylamide gel electrophoresis (SDS‒PAGE) and then transferred to a polyvinylidene difluoride (PVC) membrane (Merck KGaA, Darmstadt, Germany). The loading control and protein detection of the western blot experiment in this study was run on a single piece of gel. After membrane transfer, it was cut according to the size of the target protein. The membrane was blocked at 4 °C with 5% nonfat milk in Tris-buffered saline (TBST). The membrane was then incubated with primary antibodies and washed with TBST three times. After that, the membrane was incubated with secondary antibodies for 2 h at room temperature. Finally, Tanon™ High-sig ECL Western blotting Substrate (Tanon, Shanghai, China) was applied to the membrane to visualize the band density according to the manufacturer’s instructions. Using ImageJ image processing software (ImageJ, National Institutes of Health, USA), the bands were quantified according to their density and evaluated.

### Molecular docking

All 5 genes from the “compound-target network” were searched in the UniProtKB database (https://www.uniprot.org/) to identify relevant proteins. These proteins were searched in BLAST, and the respective protein structures were retrieved from the Protein Data Bank (PDB). The 3D conformations of the top 5 active components were downloaded from PubChem. Ligands were prepared by optimizing the energy using the MMFF94 forcefield. The proteins were cleaned, associated ligands in the crystal structure were removed, and the SwissPDB viewer was used for energy minimization. Further steps to prepare the protein were performed by AutoDock Tool software (ADT4). After adding hydrogens and neutralizing charges, 5 proteins were docked with 5 ligands each using AutoDock Vina software.

### Statistical analysis

All experiments were carried out in triplicate. Results are expressed as mean ± standard deviation. Comparisons were executed using the one-way analysis of variance (ANOVA) method with Waller-Duncan test as the post-hoc comparison method in SPSS (IBM corp. version 21). Below 0.05 p values were considered statistically significant.

## Results

### Influence of PREO on RAW 264.7 cell viability

**Fig. 1:**
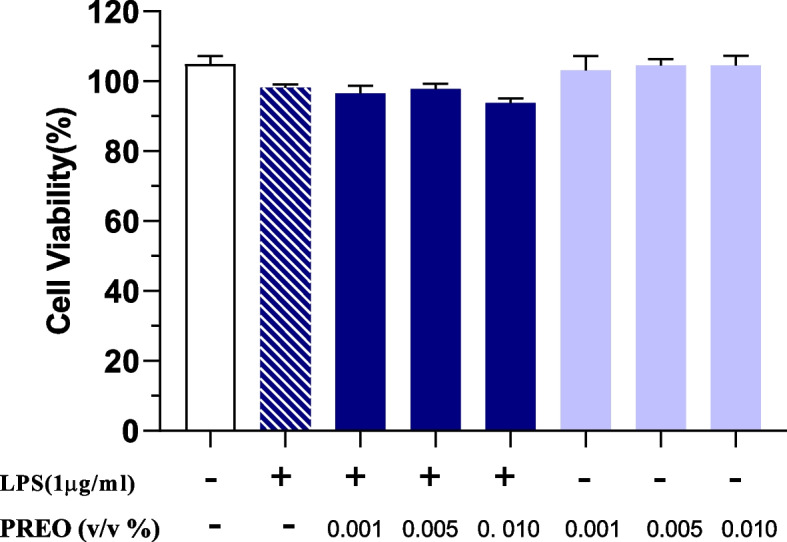
Effects of LPS and PREO on cell viability of RAW 264.7 cells. RAW 264.7 cells incubated with LPS (1 μg/mL) and different concentrations of PREO (0.001–0.010%, v/v) for 12 h. The MTT assay was used to determine cell viability. Data are normalized against the untreated control and presented as the means ± SDs (*n* = 6) of six independent experiments

The effects of PREO (0.001–0.010%) and LPS (1 μg/mL) on the viability of RAW 264.7 cells were tested using the MTT assay. The results showed that the tested doses of LPS and PREO did not affect cell viability, which remained greater than 90% (Fig. 1). Thus, 1 μg/mL LPS and 0.001–0.010% (v/v) PREO were selected for the subsequent studies.

## PREO can attenuate NO and ROS production in LPS-treated RAW 264.7 cells

**Fig. 2:**
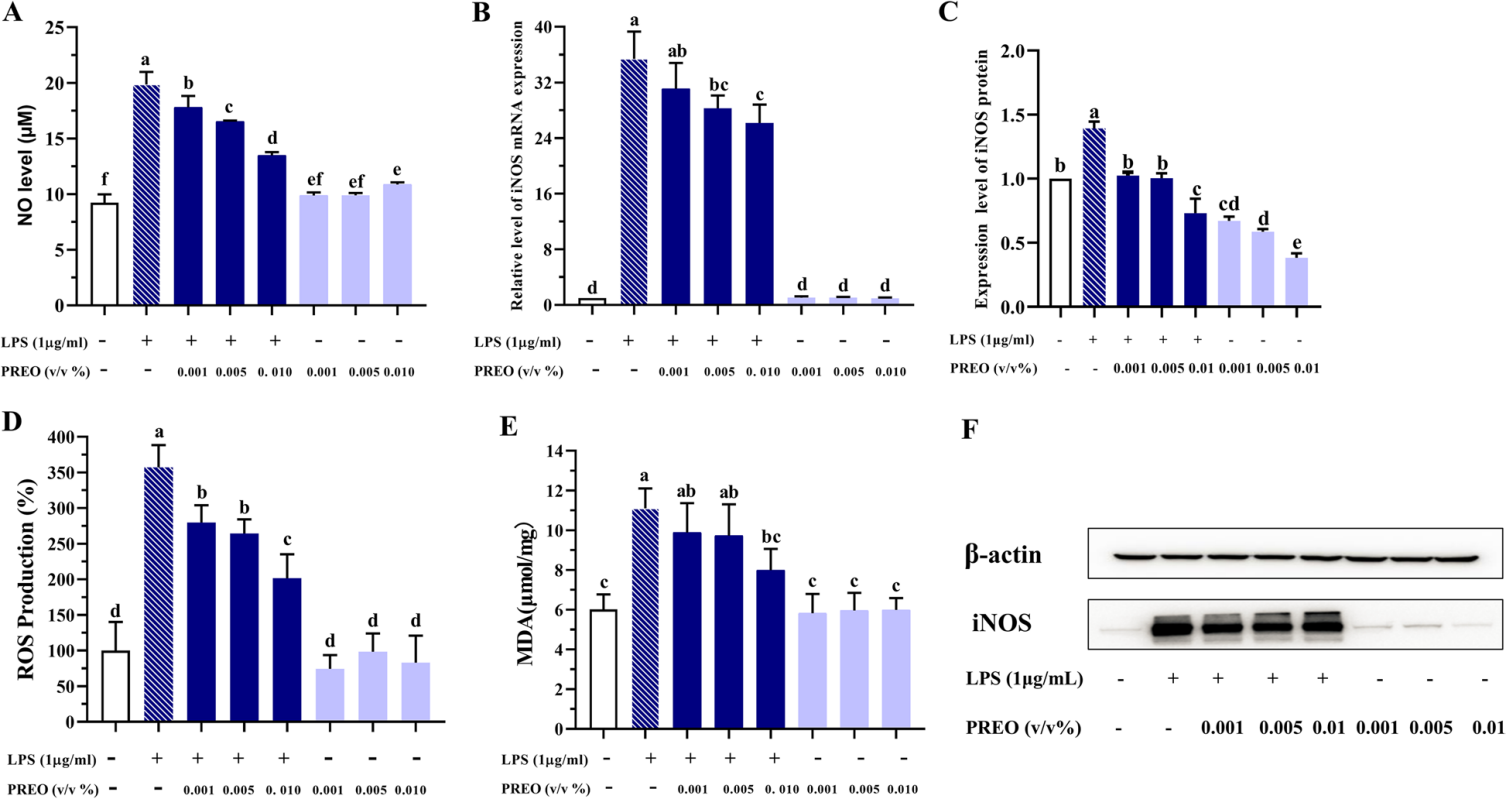
Effects of PREO on the production of NO, ROS and MDA and the expression of iNOS at the mRNA and protein levels. PREO significantly lowered the production of oxidative stress mediators in LPS-induced RAW 264.7 cells. **A:** The content of NO in cell culture medium; **B:** mRNA expression of iNOS was detected by RT‒PCR; **C:** Western blot result of iNOS protein; **D:** The intracellular reactive ROS level; **E:** Level of MDA in the cell; **F:** Western blot bands of iNOS.: Band pictures have been cropped (see supplementary data for uncropped version). Values represent means ± SDs (*n* = 3). Statistical analysis was performed by one-way ANOVA with a Waller-Duncan test. **a**-**f** Superscript with different letters denotes significant differences (*p* < 0.05) between LPS and each sample in pairs

NO and ROS are not only important inflammatory mediators but also key factors that promote the occurrence and development of oxidative stress. iNOS is an enzyme necessary to promote NO production. The production of NO and ROS was determined by the Griess method and DCFH-DA fluorescent probe method, respectively, while the mRNA and protein expression of iNOS was determined by RT‒PCR and western blotting. As shown in Fig. 2, PREO treatments exhibited no significant effect on the NO and ROS levels along with iNOS expression compared with the control. LPS (1 μg/mL) induced high production of NO and ROS and significantly increased the expression of iNOS at both the mRNA and protein levels compared to the control group. However, with the 12 h addition of PREO, all iNOS, NO, and ROS levels were significantly downregulated (*p* < 0.05) in a dose-dependent manner.

### PREO significantly influences the activities of SOD and CAT and the production of MDA

Excessive oxidative stress caused by ROS triggers the peroxidation of membrane lipids, increasing MDA formation. As a stable product of lipid peroxidation, MDA was used as a biological marker of oxidative stress. While PREO alone did not affect MDA production, it reduced the production of MDA, which increased under LPS treatment. (Fig. 2E). The increase in MDA secretion induced by LPS treatment was significantly inhibited by additional 0.1% PREO (*p* < 0.05).

**Fig. 3:**
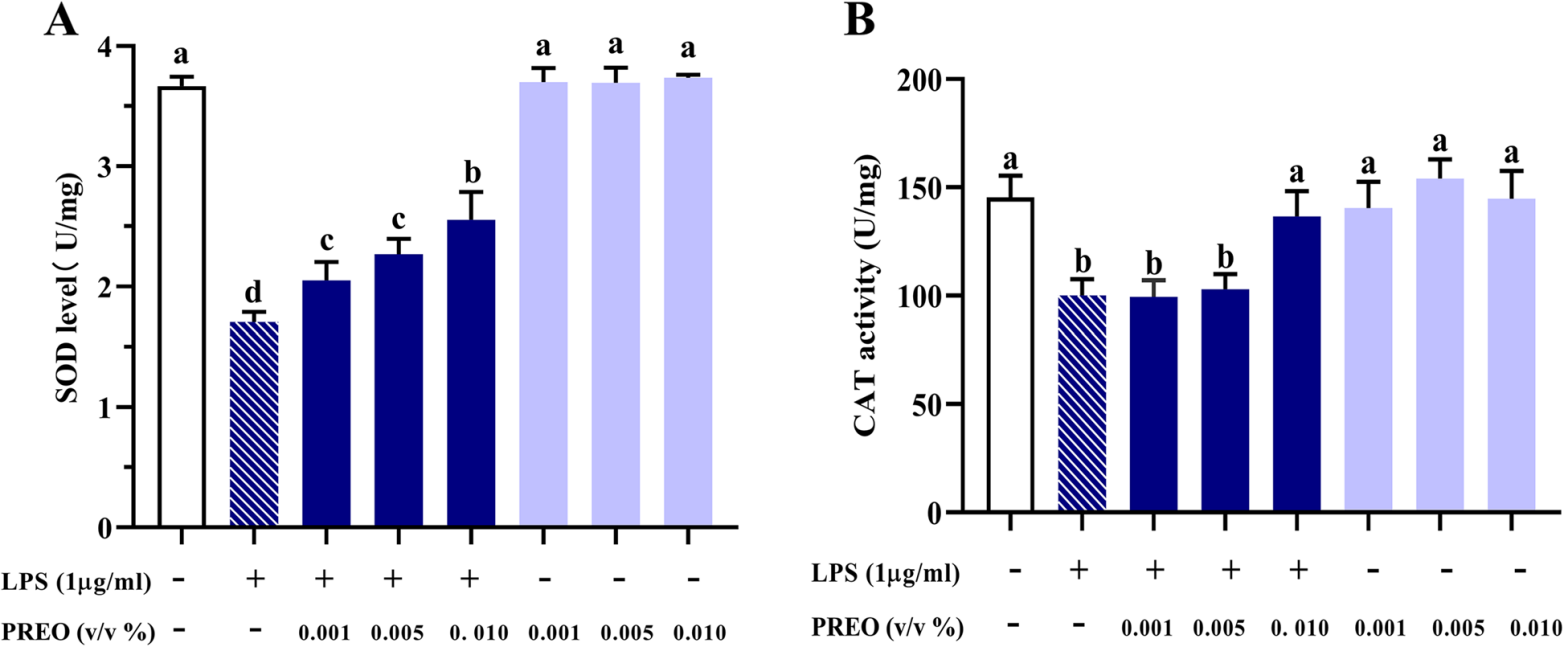
Effects of PREO on the activities of SOD and CAT. PREO increased SOD and CAT activity in LPS-treated RAW 264.7 cells. **A:** SOD activity; **B:** CAT activity. Values represent means ± SDs (*n* = 3). Statistical analysis was performed by one-way ANOVA with a Waller-Duncan test. **a**-**d** Superscripts with different letters denote significant differences (*p* < 0.05) between LPS and each sample in pairs

Antioxidant enzymes such as SOD and CAT play an important role in reducing ROS/RNS levels as a part of the antioxidation system. As shown in Fig. 3, in RAW 264.7 cells, PREO alone did not decrease the activities of SOD and CAT. However, their activities were significantly decreased (*p* < 0.05) after treatment with LPS, and 12 h of treatment with 0.001–0.1% PREO significantly enhanced the activities (*p* < 0.05). Notably, 0.01% PREO increased (*p* < 0.05) the CAT activity to the same level as the control.

#### PREO significantly reduces inflammatory cytokines production and mRNA expressions in LPS-treated RAW 264.7 cells

**Fig. 4:**
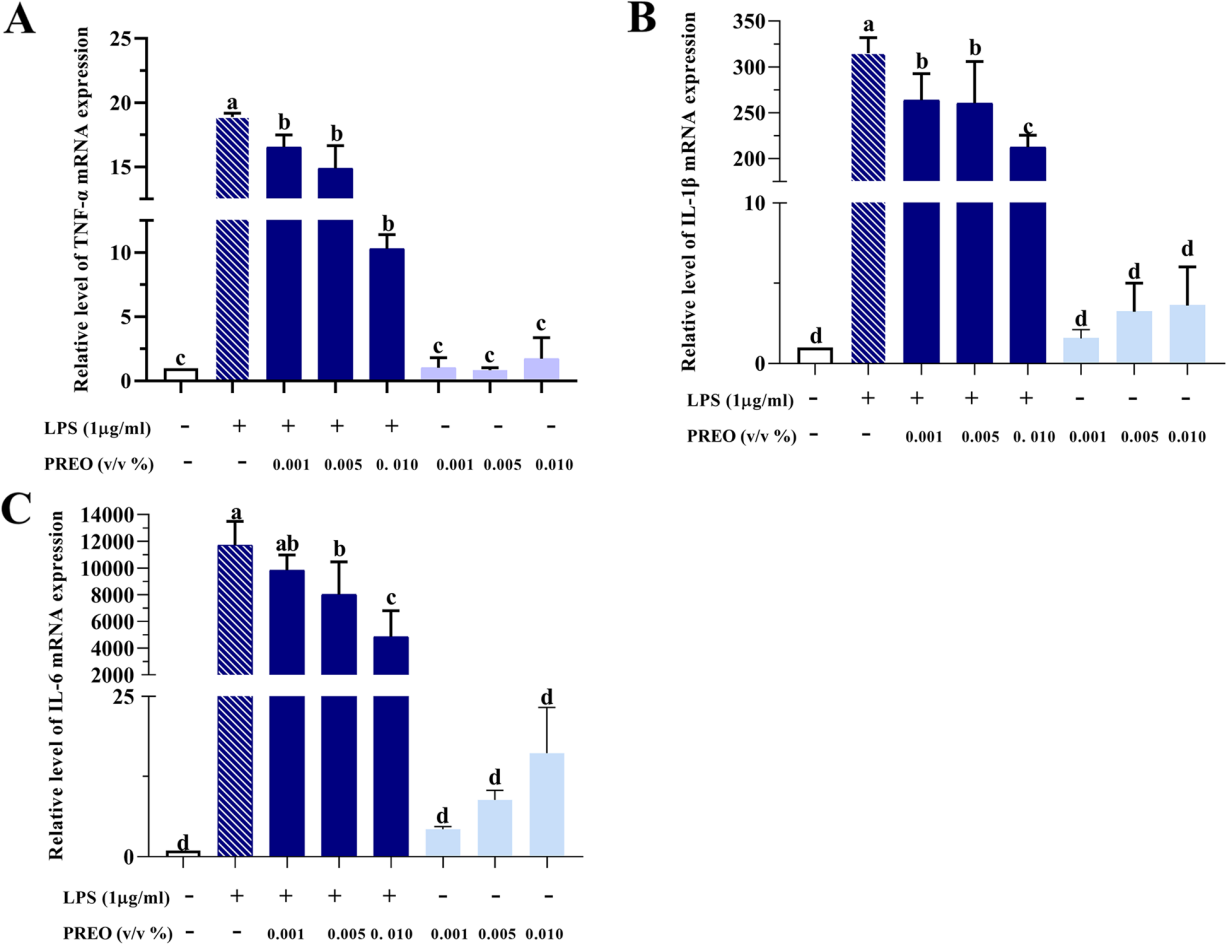
Effects of PREO on the mRNA expression of proinflammatory cytokines. PREO inhibits LPS-induced mRNA levels of TNF-α, IL-1β, and IL-6 in RAW 264.7 cells **A-C**. Values represent means ± SDs (*n* = 3). Statistical analysis was performed by one-way ANOVA with a Waller-Duncan test. a-d Superscripts with different letters denote significant differences (*p* < 0.05) between LPS and each sample in pairs

TNF-α, IL-1β, and IL-6 are common proinflammatory cytokines. As shown in Fig. 4, PREO treatment did not affect the transcription levels of TNF-α, IL-1β, and IL-6 without LPS treatment. After treatment with LPS, the expression of those proinflammatory cytokines increased and was significantly downregulated by PREO (*p* < 0.05) in a dose-dependent manner.

### GC‒MS analysis of PREO

The chemical profiling of PREO was performed using GC–MS. Fifty-seven compounds were identified and are listed in Table 2, along with the retention indexes. Citronellol and geraniol were the major components (57.34% and 9.26%, respectively). The other major compounds were pentacosane (4.66%), cis-geraniol (4.20%), methyl eugenol (3.99%), citronellol acetate (3.35%), heneicosane (2.26%), α-farnesene (2.01%), tridecanone (1.85%), eugenol (1.40%), β-linalool (1.36%), β-phenethyl alcohol (1.28%), and geranyl acetate (1%).

**Table 2:** Main compounds detected in PREO through GC‒MS analysis

No	RI	Compounds	Class	Relative peak area (%)
1	1028	α-Pinene	Monoterpene	0.73
2	1112	β-Pinene	Monoterpene	0.10
3	1124	2,4(10)-Thujadiene	Monoterpene	0.02
4	1161	β-Myrcene	Monoterpene	0.06
5	1199	L-Limonene	Monoterpene	0.13
6	1275	o-Cymene	Monoterpene	0.04
7	1280	Terpinolene	Monoterpene	0.05
8	1365	Rose oxide	Monoterpene	0.91
9	1367	trans-Rose oxide	Monoterpene	0.32
10	1429	Perillen	Monoterpene	0.02
11	1444	p-Cymenene	Monoterpene	0.02
12	1753	Geranyl acetate	Monoterpene	1.00
13	2169	Eugenol	Monoterpene	1.40
14	1231	2-Pentylfuran	Heteroaromatic	0.03
15	1282	trans-2-(2-Pentenyl) furan	Heteroaromatic	0.02
16	1413	Rosefuran	Heteroaromatic	0.01
17	1213	Eucalyptol	Monoterpenoid	0.02
18	1250	trans-β-Ocimene	Monoterpenoid	0.07
19	1252	β-Ocimene	Monoterpenoid	0.07
20	1469	Nerol oxide	Monoterpenoid	0.05
21	1514	Cyclohexane	Monoterpenoid	0.24
22	1547	β-Linalool	Monoterpenoid	1.36
23	1765	Citronellol	Monoterpenoid	54.37
24	1797	Nerol	Monoterpenoid	4.20
25	1847	Geraniol	Monoterpenoid	9.26
26	1495	Daucene	Sesquiterpene	0.20
27	1579	trans-α-Bergamotene	Sesquiterpene	0.28
28	1586	β-Copaene	Sesquiterpene	0.21
29	1661	α-Himachalene	Sesquiterpene	0.58
30	1727	β-Bisabolene	Sesquiterpene	0.17
31	1735	Bicyclogermacren	Sesquiterpene	0.85
32	1746	α-Farnesene	Sesquiterpene	2.01
33	2215	α-Bisabolol	Sesquiterpene	0.67
34	2350	Farnesol	Sesquiterpene	0.40
35	1320	2-Heptanol	Fatty alcohols	0.05
36	1450	1-Octene-3-ol	Fatty alcohols	0.01
37	1615	Citronellyl formate	Fatty alcohols	0.15
38	1660	Citronellol acetate	Fatty alcohols	3.35
39	1903	2-Tridecanol	Fatty alcohols	0.15
40	2165	1-Tetradecanol	Fatty alcohols	0.14
41	1338	5-Hepten-2-one	Ketone	0.02
42	1465	6-Methyl-5-hepten-2-ol	Ketone	0.01
43	1598	Methyl nonyl ketone	Ketone	0.26
44	1809	Tridecanone	Ketone	1.85
45	2123	Cyclododecanol	Ketone	0.07
46	2217	Pentacosane	Ketone	4.66
47	1435	Ethyl octanoate	Ester	0.02
48	1813	Phenethyl acetate	Ester	0.24
49	2098	Tetradecanol acetate	Ester	0.56
50	2100	Heneicosane	Alkane	2.26
51	2108	Pentadecanol	Alkane	0.04
52	1184	Heptanal	Aldehydes	0.12
53	1639	Isosativene	Terpene	0.11
54	1725	Naphthalene	Paradichlorobenzene	0.30
55	1906	β-Phenethyl alcohol	Ethanol	1.28
56	2013	Methyleugenol	Terpenoid	3.99
57	2599	15-Hydroxy-α-muurolene	sesquiterpenoids	0.45
Monoterpene and aromatics				4.86
Monoterpenoid				69.64
Sesquiterpene				5.37
Fatty alcohols				3.85
Ketone				6.87
Ester and alkanes				3.12
Others				6.25
Total				99.96

### Network pharmacology

#### Main active components and druggable targets of PREO

**Fig. 5:**
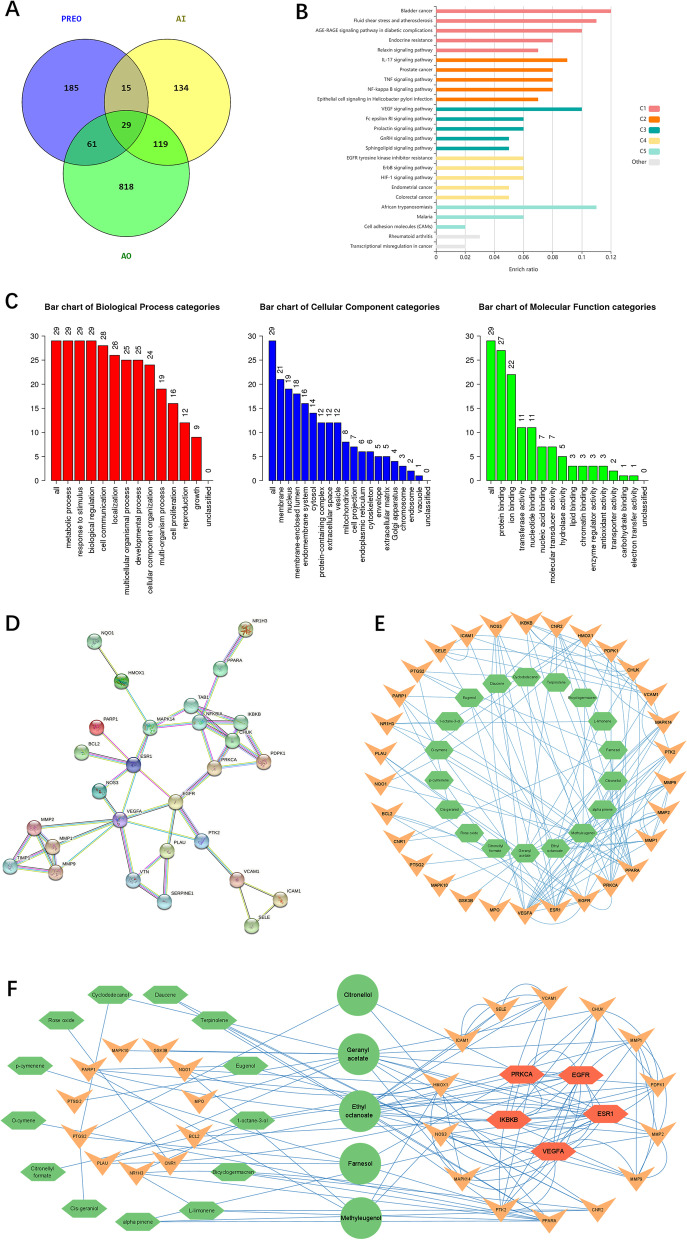
Network pharmacology analysis for the PREO compound-target network. **A:** Venn diagram of PREO components, anti-inflammation (AI), and antioxidant (AO) effects. **B:** Bar plot of pathway enrichment by Kobas 3.0; **C:** Gene Ontology (GO) analysis for biological process, cellular components, and molecular function. **D:** STRING network of protein‒protein interactions. **E:** Compound-target network: green hexagonal nodes are compounds, and orange arrow-shaped nodes are targets. **F:** Active ingredients and node-based top 5 targets depending on degree and closeness. Green circular nodes are the top 5 active ingredients, and bright orange hexagonal nodes are the top 5 targets

From the 57 components identified by the GC‒MS results, 24 components were determined to be active depending on (oral bioavailability) OB% and CaCO-2 permeation. Upon analyzing their respective targets for anti-inflammatory and antioxidative actions, 5 compounds were selected: citronellol (compound ID = 8842), ethyl octanoate (compound ID = 7799), farnesol (compound ID = 445,070), geraniol acetate (compound ID = 1,549,026) and methyl eugenol (compound ID = 7127) (Table 3). After eliminating the duplicate values, 544 gene targets related to PREO compounds were obtained from the Swiss-Target-Prediction database. A total of 1324 gene targets related to AI and AO were collected from the Gene Cards database. After interaction analysis, 29 gene targets were identified by considering the interaction between the targets of AO, AI, and PREO, as shown in Fig. 5A.

**Table 3:** Active ingredients of PREO depending on OB%

Name	Structure	Pubchem ID	Molecular formula	Molecular weight (g/mol)	OB%	CaCO-2 permeation
Methyl eugenol		7127	C_11_H_14_O_2_	178.25	73.36	1.47
Citronellol		8842	C_10_H_20_O	156.3	38.05	1.19
Ethyl octanoate		7799	C_10_H_20_O_2_	172.3	33.05	1.25
Farnesol		445,070	C_15_H_26_O	222.4	28.44	1.32
Geranyl acetate		1,549,026	C_12_H_20_O_2_	196.32	25.4	1.28

The data of active components and 29 targets were imported into Cytoscape v3.7.1 software to construct the “active component-target” network of PREO in the treatment of inflammation, and the network topology parameters were calculated by network analysis. There were 117 connections between 24 compounds and 29 targets (Fig. 5E). Based on the highest confidence score (> 0.9), 21 protein targets formed a PPI network with 60 edges, as presented in Fig. 5D. Further cytoNCA analysis shown in Fig. 5F indicated that EGFR (degree = 10), ESR1 (degree = 10), IKBKB (degree = 6), PKRCA (degree = 8) and VEGFA (degree = 14) might be the main interaction targets of the components.

### GO and KEGG enrichment analysis

Based on GO biological process analysis, all 29 genes were involved in metabolic processes, biological regulation, and the response to stimulus, and 16 genes were involved in cell proliferation. In the GO cellular localization category, 21 genes encoded proteins located in the cell membrane, while 19 genes encoded proteins present in the nucleus. Regarding GO molecular function, 27 genes were found to be related to protein binding, 21 genes to ion binding, and 3 genes each to enzyme-related activity, lipid binding and antioxidant activity (Fig. 5C). According to KEGG enrichment analysis, the most prominent inflammatory pathways were IL-17, NF-κB and TNF signaling (Fig. 5B).

### PREO decreases NF-κB production by reducing the phosphorylation of related proteins

**Fig. 6:**
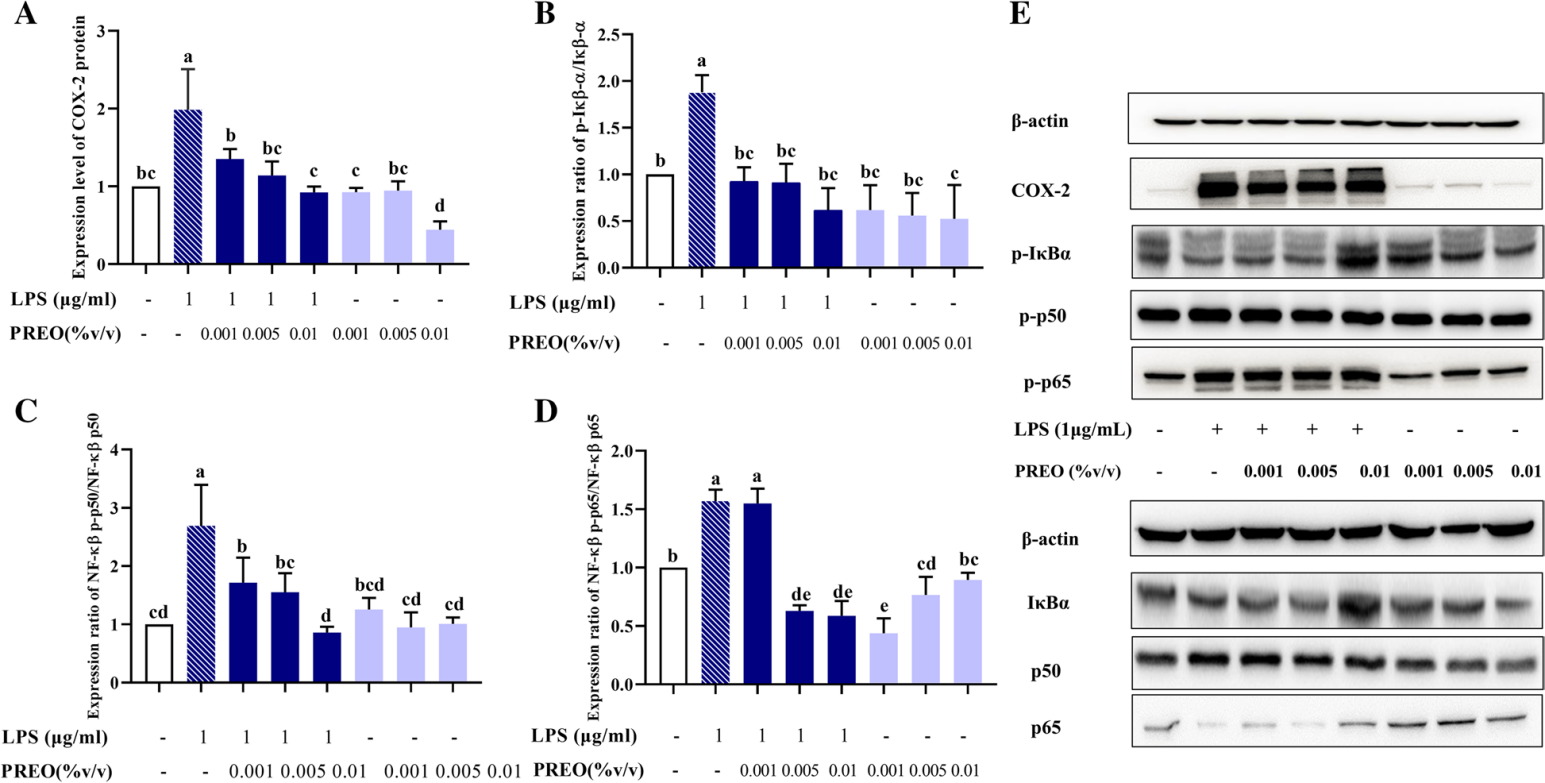
Effects of PREO on protein expression that are involved in the NF-κB inflammatory pathway in LPS-stimulated RAW 264.7 cells. PREO decreased the expression of inflammatory pathway proteins. **A:** COX-2. **B:** p-IκB-α/IκB-α. **C:** NF-κB p-p50/NF-κB p50. **D:** NF-κB p-p65/NF-κB p65. **E:** Western blot bands for the proteins. The final imaged band is not an entire piece of gel, but rather each particular protein that has been incubated. The bands have been cropped to adjust the size. See supplementary data for uncropped version. Statistical analysis was performed by one-way ANOVA with a Waller-Duncan test. The “a-e” superscripts denote pairwise significant differences (*p* < 0.05) between LPS and each sample

**Fig. 7:**
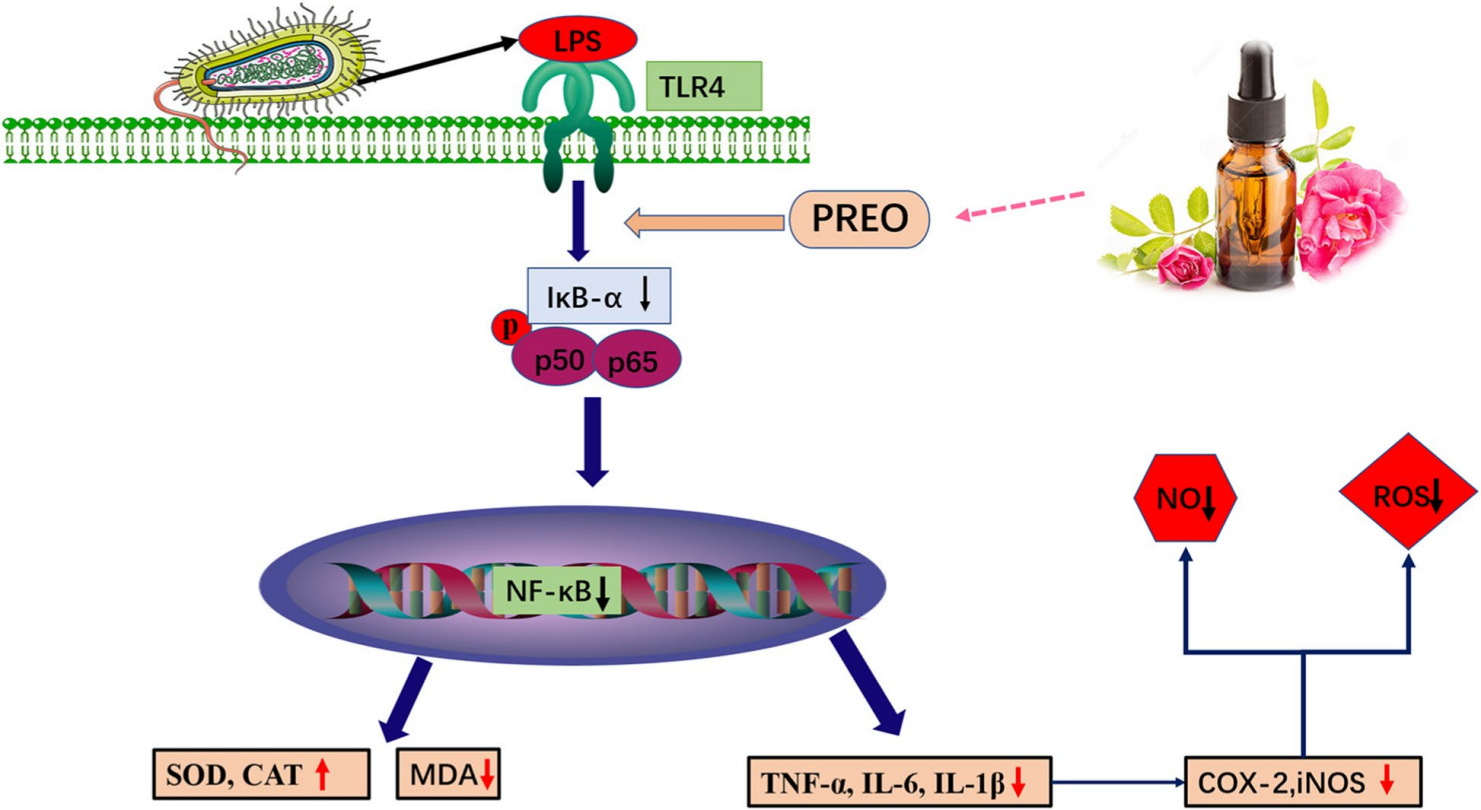
A schematic of the proposed mechanism by which PREO inhibits LPS-stimulated inflammation in RAW 264.7 cells

According to our results presented in Fig. 6, LPS markedly increased the expression of p65, p50, and IκB-α (*p* < 0.05) and promoted their phosphorylation. PREO treatments significantly reduced the expression of p65, p50, and IκB-α and inhibited the phosphorylation of p65, p50, and IκB-α in macrophage cells (*p* < 0.05). In addition, PREO significantly reduced the protein expression of COX-2 (*p* < 0.05). These experimental findings indicate that PREO can mediate inflammation in macrophage cells induced by LPS through NF-κB pathways (Fig. 7).

## Molecular docking

**Fig. 8:**
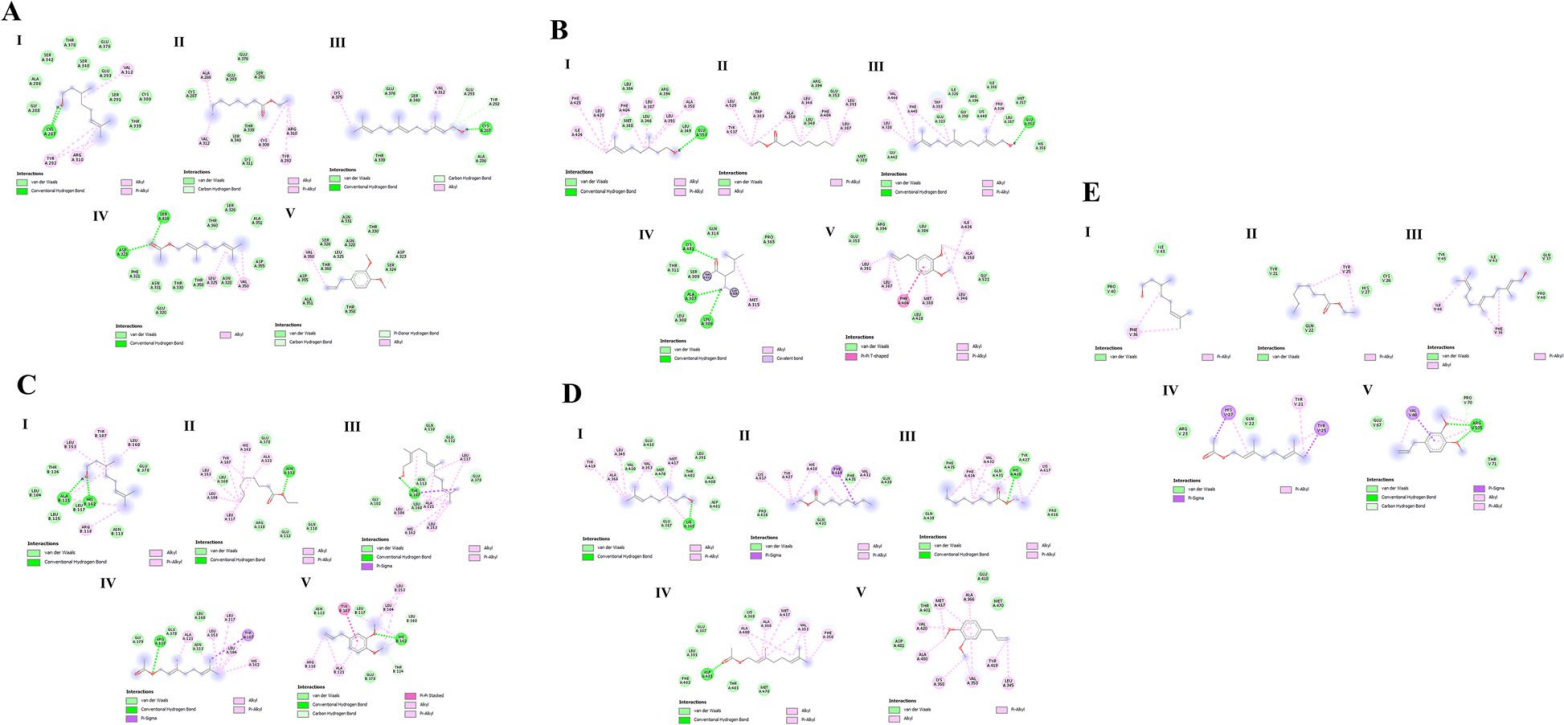
Predicted interactions between PREO components as ligands and inflammatory proteins. **A (I-V):** EGFR interactions with citronellol, ethyl octanoate, farnesol, geranyl acetate and methyl eugenol; **B (I-V):** ESR1 interactions with citronellol, ethyl octanoate, farnesol, geranyl acetate and methyl eugenol; **C (I-V):** IKBKB interactions with citronellol, ethyl octanoate, farnesol, geranyl acetate and methyl eugenol; **D (I-V):** PRKCA interactions with citronellol, ethyl octanoate, farnesol, geranyl acetate and methyl eugenol; **E (I-V):** VEGFA interactions with citronellol, ethyl octanoate, farnesol, geranyl acetate and methyl eugenol

As shown in Table 4 and Fig. 8, among the 5 compounds chosen, geranyl acetate showed the best interaction with EGFR, ESR1, and PRKCA with the smallest energies of -5.5 kcal/mol, -5.8 kcal/mol, and -5.4 kcal/mol, respectively. Methyl eugenol interacted with IKBKB and farnesol, with VEGFA showing the lowest binding affinities among the other components (-5.8 kcal/mol and -4.1 kcal/mol, respectively). In all the docked models, all ligands interacted best with IKBKB and ESR1. However, citronellol exhibited closer interaction affinities on average than the others.

**Table 4:** Molecular docking affinities with ligands and human proteins

Protein	Compounds	Binding affinity ((kcal/mol)	Interaction bonds	
			**H-bonds/Alkyl bonds/Covalent bonds**	**van der Waals**
EGFR	Citronellol	-4.9	Cys287, Tyr292, Val312, Arg310	Ala286, Gly288, Ser291, Glu293, Cys309, Thr339, Ser340, Ser342, Glu376, Thr378
	Ethyl octanoate	-4.3	Ala286, Tyr292, Cys309, Arg310, Val312, Ser340	Cys287, Ser291, Glu293, Lys311, Thr339, Glu376
	Farnesol	-5.4	Cys287, Tyr292, Glu293, Val312, Lys375	Ala286, Thr339, Ser 340, Glu 376
	Geranyl acetate	-5.5	Leu325, Val 350, Asp323, Ser324,	Glu320, Phe321, Ser326, Asn328, Thr330, Asn331, Ala351, Thr358, Thr360
	Methyl-eugenol	-5.2	Asp323, Leu325, Val350	Ser324, Ser326, Asn328, Thr330, Asn331, Ala351, Asp355, Thr358, Thr360
ESR1	Citronellol	-5.4	Ala350, Glu353, Leu387, Leu391, Phe404, Ile424, Phe425, Leu428	Leu346, Leu349, Leu384, Met388, Arg394
	Ethyl octanoate	-5.0	Leu346, Ala350, Trp383, Leu387, Leu391, Phe404, Leu525, Tyr537	Met343, Leu349, Glu353, Met388, Arg394
	Farnesol	-5.7	Leu320, Pro324, Glu353, Trp393, Phe445, Val446	Glu323, Ile326, His356, Met357, Ile386, Leu387, Gly390, Arg394, Gly442, Lys449
	Geranyl acetate	-5.8	Leu346, Ala350, Leu387, Leu391, Phe404	Met343, Leu349, Glu353, Leu384, Met388, Arg394, Ile424, Phe425, Gly521, Leu525
	Methyl-eugenol	-5.6	Leu346, Ala350, Leu387, Met388, Leu391, Phe404, Ile424	Glu353, Leu384, Arg394, Leu428, Gly521
IKBKB	Citronellol	-5.4	Tyr107, Arg118, Ala121, Leu153, Leu160, His162	Leu104, Asn113, Leu117, Thr124, Leu125, Glu378
	Ethyl octanoate	-4.9	Leu104, Tyr107, Asn113, Leu117, Ala121, Leu153, His162	Gln110, Glu112, Arg118, Leu160, Glu378
	Farnesol	-5.7	Leu104, Asn113, Leu117, Ala121, Leu153, His162	Gly101, Gln110, Glu112, Asn113, Leu160, Glu378
	Geranyl acetate	-5.5	Leu104, Tyr107, Leu117, Arg118, Ala121, Leu153, His162	Asn113, Leu160, Glu378, Gly379
	Methyl-eugenol	-5.8	Leu104, Tyr107, Arg118, Ala121, Thr124, Leu153, Leu160, His162	Asn113, Leu117, Glu378
PRKCA	Citronellol	-4.9	Leu345, Val353, Ala366, Lys368, Met417, Tyr419	Glu387, Leu391, Thr401, Glu418, Val420, Met470, Ala480, Asp481
	Ethyl octanoate	-4.8	Tyr427, His428, Val432, Phe614, Lys617	Gln431, Phe435, Gln439, Pro616
	Farnesol	-4.6	His428, Gln431, Val432, Phe614, Lys617	Tyr427, Gln431, Phe435, Gln439, Pro616
	Geranyl acetate	-5.4	Phe350, Val353, Ala366, Met417, Ala480, Asp481	Lys368, Glu387, Leu391, Thr401, Met470, Phe482
	Methyl-eugenol	-5.3	Leu345, Val353, Ala366, Lys368, Met417, Tyr419, Val420, Ala480	Thr401, Glu418, Met470, Asp481
VEGFA	Citronellol	-3.3	Phe36	Pro40, Ile43
	Ethyl octanoate	-2.8	Tyr25	Tyr21, Gln22, Cys26, His27
	Farnesol	-4.1	Phe36, Ile46	Gln37, Pro40, Ile43, Tyr45
	Geranyl acetate	-4.0	Tyr21, Tyr25, His27	Gln22, Arg23
	Methyl-eugenol	-3.6	Val69, Pro70, Arg105	Glu67, Thr71

## Discussion

Rose is not only a popular horticultural ornamental plant for aromatic environment construction but also a widely used food flavoring agent and cosmetic additive. There are over 200 species and more than 1800 cultivars. However, only a few species (*R. damascena*, *R. centifolia*, *R. gallica*, *R. alba,* and *R. rugosa*) are used for essential oil (EO) extraction. Through GC‒MS investigation, 57 compounds (> 0.01%) found in the PREO could be classified into 8 groups. Monoterpenoids (69.64%) were the main group, followed by ketones (6.87%), sesquiterpenes (5.37%), fatty alcohols (3.85%), esters and alkanes (3.12%) as well as others (6.25%). Beta-phenyl ethyl alcohol, citronellol, geraniol, eugenol, linalool, and rose oxide are common chemicals found in rose-based extracts, including REOs [24]. However, the compositions of REOs from different parts of the world vary considerably. For example, *R. damascena* EO from Pakistan contains 70.9% *β*-phenyl ethyl alcohol. The percentage ranges of other components, such as citronellol (20–34%), geraniol (5–22%), and nerol (5–12%), also differ based on their regional origin [19, 25]. In our study, the percentages of citronellol, geraniol, nerol, β-phenyl ethyl alcohol and rose oxide in PREO were 57.34%, 9.26%, 4.2%, 1.28 and 0.91%, respectively.

As shown in this study, PREO inhibited the production of NO, ROS, and MDA and exhibited a stronger effect on SOD and CAT promotion in a concentration-dependent manner. PREO is a complex solution, and the antioxidative and anti-inflammatory effects may be correlated with the synergistic function of the chemical components. Hence, to determine the possible active components from this mixture, we performed network pharmacological studies. Network pharmacology is an analysis tool to determine target-compound relationships for drug development. Twenty-four components were identified as more active ingredients through network pharmacology analysis for “*Homo sapiens*”. The transcription factor NF-κB regulates multiple aspects of innate and adaptive immune functions and serves as a pivotal mediator of inflammatory responses. LPS stimulation can activate pathways such as NF-κB and promote proinflammatory cytokine overexpression, consequently inducing oxidative stress. Phosphorylation of the NF-κB pathway proteins p50, p65, and IκBα increased due to LPS induction, which was consistent with other studies [26]. p50 and p65 dimer disassociated from IκBα and induce the production of COX-2, iNOS and other proinflammatory cytokines. PREO showed significant regulatory effects on the phosphorylation of p50, p65, and IκBα in the NF-κB pathway, resulting in notable decreases in the mRNA expression of TNF-α, IL-1β, IL-6, COX-2, and iNOS., All these results suggested that PREO could effectively ameliorate the inflammation and oxidative stress of macrophage cells induced by LPS in vitro. According to other studies, REOs from *R. damascene* [27], *Pelargonium graveolens* [28] and *Rosmarinus officinalis* [29] also showed good anti-inflammatory effects in animal models, but further mechanistic investigation was not reported. Anti-inflammatory effects by regulating the MAPK and NF-κB pathways in LPS-treated RAW 264.7 cells were found in other essential oils, such as oregano EOs [30].

However, the amount of citronellol (54.3%) in PREO was higher than in any other REO. Using relatively low energy, citronellol showed better interactions with EGFR, ESR1, IKBKB, PRKCA, and VEGFA, which are the key promotors of inflammatory pathways such as MAPK and NF-κB signaling. It might be suggested that citronellol could interact with the pathways to regulate inflammation. From previous investigations on the bioactivities of citronellol, it appeared to be a strong anti-inflammatory agent. Citronellol from geranium essential oil inhibited cytokine production in mast cells [31]. Melo et al. 2011 showed in their investigation with a Swiss mouse model that citronellol has anti-inflammatory and redox-protective functions [32]. In another in vitro experiment with LPS-induced macrophage cells and in an in vivo rodent model, citronellol treatment reduced oxidative stress and inhibited inflammation significantly [33]. We hypothesized that citronellol from PREO may have played a crucial role in downregulating the NF-κB pathway in our experiment.

## Conclusion

PREO exerted an inhibitory effect on inflammation and oxidative stress and might ultimately act through the downstream NF-κB pathway. Network pharmacology and molecular docking revealed that citronellol might be involved in the interaction with key proteins in the NF-κB pathway. These results indicated that PREO is a potent anti-inflammatory and antioxidative agent. However, whether components such as citronellol from PREO participate in the pathway regulation should be investigated further, and the mechanism should be elucidated.

## Data Availability

The data used to support the findings of this study are available from the corresponding author upon request.

## Abbreviations

TCM: Traditional Chinese medicine
PREO: Pingyin rose essential oil
LPS: Lipopolysaccharides
MDA: Malondialdehyde
SOD: Superoxide dismutase
CAT: Catalase
ROS: Reactive oxygen species
NO: Nitric oxide
TLR4: Toll-like receptor 4
NF-Κb: Nuclear factor kappa B:
TNF-α,: Tumor necrosis factor alpha
IL: Interleukin
REO: Rose essential oil
GLP-1: Glucagon like peptide 1
DMEM: Dulbecco's modified Eagle medium
FBS: Fetal bovine serum
DCFH_2_-DA: Dichlorofluorescein-diacetate
BCA: Bicinchoninic acid
GC‒MS: Gas chromatography-coupled to mass spectrometry
SDS: Sodium dodecyl sulfate

## References

[1] Zhang Y, Guo H, Cheng BCY, Su T, Fu XQ, Li T (2018). Dingchuan tang essential oil inhibits the production of inflammatory mediators via suppressing the IRAK/NF-κB, IRAK/AP-1, and TBK1/IRF3 pathways in lipopolysaccharide-stimulated RAW264.7 cells. Drug Design, Dev Ther.

[2] Polednik KM, Koch AC, Felzien LK (2018). Effects of essential oil from thymus vulgaris on viability and inflammation in Zebrafish embryos. Zebrafish.

[3] Duque GA, Descoteaux A (2014). Macrophage cytokines: Involvement in immunity and infectious diseases. Front Immunol.

[4] Medzhitov R (2008). Origin and physiological roles of inflammation. Nat.

[5] Aviello G, Knaus UG (2017). ROS in gastrointestinal inflammation: rescue or sabotage?. Br J Pharmacol.

[6] Bhattacharyya A, Chattopadhyay R, Mitra S, Crowe SE. Oxidative stress: an essential factor in the pathogenesis of gastrointestinal mucosal diseases. Physiological Reviews. 2014;94(2):329-54. 10.1152/physrev.00040.2012 PMC4044300 24692350

[7] Hinz M, Scheidereit C (2014). The IκB kinase complex in NF-κB regulation and beyond. EMBO Rep.

[8] Wongrakpanich S, Wongrakpanich A, Melhado K, Rangaswami J (2018). A comprehensive review of non-steroidal anti-inflammatory drug use in the elderly. Aging Dis.

[9] Borges RS, Ortiz BLS, Pereira ACM, Keita H, Carvalho JCT (2019). Rosmarinus officinalis essential oil: A review of its phytochemistry, anti-inflammatory activity, and mechanisms of action involved. J Ethnopharmacol.

[10] Ho CL, Li LH, Weng YC, Hua KF, Ju TC (2020). Eucalyptus essential oils inhibit the lipopolysaccharide-induced inflammatory response in RAW264.7 macrophages through reducing MAPK and NF-κB pathways. BMC Complement Med Ther.

[11] Kuttan R, Jeena K, Liju VB (2013). Antioxidant, anti-inflammatory and antinociceptive activities of essential oil from ginger. Indian J Physiol Pharmacol..

[12] Valente J, Zuzarte M, Gonçalves MJ, Lopes MC, Cavaleiro C, Salgueiro L (2013). Antifungal, antioxidant and anti-inflammatory activities of Oenanthe crocata L. essential oil. Food Chem Toxicol.

[13] Shen CY, Jiang JG, Zhu W, Ou-Yang Q (2017). Anti-inflammatory effect of essential oil from citrus aurantium L. var. amara Engl. J Agri Food Chem.

[14] Jafari M, Zarban A, Pham S, Wang T (2008). Rosa damascena decreased mortality in adult drosophila. J Med Food.

[15] Gholamhoseinian A, Fallah H, Sharifi far F (2009). Inhibitory effect of methanol extract of Rosa damascena Mill. flowers on α-glucosidase activity and postprandial hyperglycemia in normal and diabetic rats. Phytomed.

[16] Uysal M, Doğru HY, Sapmaz E, Tas U, Çakmak B, Ozsoy AZ (2016). Investigating the effect of rose essential oil in patients with primary dysmenorrhea. Complement Ther Clin Pract.

[17] Verma A, Srivastava R, Sonar PK, Yadav R (2020). Traditional, phytochemical, and biological aspects of Rosa alba L.: a systematic review. Fut J Pharm Sci.

[18] Almasirad A, Amanzadeh Y, Taheri A, Iranshahi M (2007). Composition of a historical rose oil sample (rosa damascena mill., rosaceae). J Essent Oil Res.

[19] Mahboubi M (2016). Rosa damascena as holy ancient herb with novel applications. J Tradit Complement Med.

[20] Xie Y, Zhang W (2012). Antihypertensive activity of Rosa rugosa thunb. flowers: angiotensin i converting enzyme inhibitor. J Ethnopharmacol.

[21] Ng TB, He JS, Niu SM, Pi ZF, Shao W, Liu F (2010). A gallic acid derivative and polysaccharides with antioxidative activity from rose ( Rosa rugosa ) flowers. J Pharm Pharmacol.

[22] Ren G, Xue P, Sun X, Zhao G (2018). Determination of the volatile and polyphenol constituents and the antimicrobial, antioxidant, and tyrosinase inhibitory activities of the bioactive compounds from the by-product of Rosa rugosa Thunb. var. plena Regal tea. BMC Complement Altern Med.

[23] Livak KJ, Schmittgen TD (2001). Analysis of relative gene expression data using real-time quantitative PCR and the 2-ΔΔCT method. Methods.

[24] Xiao Z, Luo J, Niu Y, Wu M (2018). Characterization of key aroma compounds from different rose essential oils using gas chromatography-mass spectrometry, gas chromatography–olfactometry and partial least squares regression. Nat Prod Res.

[25] Mohebitabar S, Shirazi M, Bioos S, Rahimi R, Malekshahi F, Nejatbakhsh F (2017). Therapeutic efficacy of rose oil: a comprehensive review of clinical evidence. Avicenna J Phytomed.

[26] Li D, Liu Q, Sun W, Chen X, Wang Y, Sun Y (2018). 1,3,6,7-Tetrahydroxy-8-prenylxanthone ameliorates inflammatory responses resulting from the paracrine interaction of adipocytes and macrophages. Br J Pharmacol.

[27] Hajhashemi V, Ghannadi A, Hajiloo M (2010). Analgesic and anti-infammatory effects of rosa damascena hydroalcoholic extract and its essential oil in animal models. Iran J Pharm Res.

[28] Boukhatem MN, Kameli A, Ferhat MA, Saidi F, Mekarnia M (2013). Rose geranium essential oil as a source of new and safe anti-inflammatory drugs. Libyan J Med.

[29] Takaki I, Bersani-Amado LE, Vendruscolo A, Sartoretto SM, Diniz SP, Bersani-Amado CA (2008). Anti-inflammatory and antinociceptive effects of Rosmarinus officinalis L. essential oil in experimental animal models. J Med Food.

[30] Cheng C, Zou Y, Peng J (2018). Oregano essential oil attenuates raw264.7 cells from lipopolysaccharide-induced inflammatory response through regulating nadph oxidase activation-driven oxidative stress. Mol.

[31] Kobayashi Y, Sato H, Yorita M, Nakayama H, Miyazato H, Sugimoto K (2016). Inhibitory effects of geranium essential oil and its major component, citronellol, on degranulation and cytokine production by mast cells. Biosci Biotechnol Biochem.

[32] Melo MS, Guimarães AG, Santana MF, Siqueira RS, de Lima A do CB, Dias AS (2011). Anti-inflammatory and redox-protective activities of citronellal. Biol Res.

[33] Brito RG, Guimarães AG, Quintans JSS, Santos MRV, de Sousa DP, Badaue-Passos D (2012). Citronellol, a monoterpene alcohol, reduces nociceptive and inflammatory activities in rodents. J Nat Med.

